# Effects of modern aesthetic dental fillings on proton therapy

**DOI:** 10.1016/j.phro.2024.100552

**Published:** 2024-02-15

**Authors:** Yun Ming Wong, Calvin Wei Yang Koh, Kah Seng Lew, Clifford Ghee Ann Chua, Ping Lin Yeap, Wibawa Andrew, Master Zubin, Sharon Shuxian Poh, Wen Siang Lew, James Cheow Lei Lee, Sung Yong Park, Hong Qi Tan

**Affiliations:** a Division of Physics and Applied Physics, Nanyang Technological University, Singapore; b Division of Radiation Oncology, National Cancer Centre Singapore, Singapore; c Oncology Academic Clinical Programme, Duke-NUS Medical School, Singapore; d Department of Oncology, University of Cambridge, United Kingdom

**Keywords:** Dental fillings, Composite resin, Glass ionomer cement, SPR prediction, Dose perturbation, Proton therapy

## Abstract

**Background and purpose:**

High-density dental fillings pose a non-negligible impact on head and neck cancer treatment. For proton therapy, stopping power ratio (SPR) prediction will be significantly impaired by the associated image artifacts. Dose perturbation is also inevitable, compromising the treatment plan quality. While plenty of work has been done on metal or amalgam fillings, none has touched on composite resin (CR) and glass ionomer cement (GIC) which have seen an increasing usage. Hence, this work aims to provide a detailed characterisation of SPR and dose perturbation in proton therapy caused by CR and GIC.

**Materials and methods:**

Four types of fillings were used: CR, Fuji Bulk (FB), Fuji II (FII) and Fuji IX (FIX). The latter three belong to GIC category. Measured SPR were compared with SPR predicted using single-energy computed tomography (SECT) and dual-energy computed tomography (DECT). Dose perturbation of proton beams with lower- and higher-energy levels was also quantified using Gafchromic films.

**Results:**

The measured SPR for CR, FB, FII and FIX were 1.68, 1.77, 1.77 and 1.76, respectively. Overall, DECT could predict SPR better than SECT. The lowest percentage error achieved by DECT was 19.7 %, demonstrating the challenge in estimating SPR, even for fillings with relatively lower densities. For both proton beam energies and all four fillings of about 4.5 mm thickness, the maximum dose perturbation was 3 %.

**Conclusion:**

This study showed that dose perturbation by CR and GIC was comparatively small. We have measured and recommended the SPR values for overriding the fillings in TPS.

## 1 Introduction

Dental fillings are one of the most commonly received treatments for dental cavities. On average, American adults have three dental fillings per person [1], and 84 % of British adults with at least one tooth have a filling [2]. Given these statistics, it is expected that a considerable number of head and neck (H&N) cancer patients would have dental fillings.

While photon therapy techniques, for example, intensity-modulated radiation therapy (IMRT) and volumetric modulated arc therapy (VMAT), have long been used for H&N cancer treatment, proton therapy has emerged as a good alternative because of its dose sparing in organs at risk (OARs). Numerous studies have demonstrated reduced toxicities and osteonecrosis incidence in H&N cancers with proton therapy [3], [4], [5], [6], [7], [8]. That being said, the uncertainty of stopping power ratio (SPR) will undermine the dose calculation accuracy during proton therapy treatment planning [9]. This uncertainty arises mainly due to the inadequacy of single-energy computed tomography (SECT) Hounsfield look-up table (HLUT) to predict SPR accurately [10]. Dual‐energy computed tomography (DECT) has shown promise in this regard, improving SPR prediction by 1 – 2 % for biological tissues or tissue-equivalent materials [11], [12], [13], [14], [15], [16], and up to about 37 % for non-tissue materials [17].

Besides the challenge in estimating SPR, another major issue associated with high-Z non-tissue implant is the image artifacts resulting from various factors like beam hardening and scattering [18]. These will affect the computed tomography (CT) number accuracy in nearby soft tissues and hinder precise material contouring. One proposed solution is the metal artifact reduction algorithms [19], [20], such as iterative metal artifact reduction (iMAR) algorithm which aims to reduce metal artifacts through beam hardening correction, normalized sinogram inpainting, and frequency split [21].

Dental fillings are also known to cause a non-negligible dose perturbation which may or may not be accounted for accurately during treatment planning. A number of studies have shown that commonly used fillings such as gold and amalgam fillings cause a decrease in the downstream dose, be it for photon therapy [22], [23], [24], or proton therapy [25], [26]. As a consequence, the target coverage could potentially be reduced. To mitigate this effect, avoidance strategies are typically implemented, but this might cause the treatment plan quality to be compromised [25] and is not always possible [20] especially in the case of treatment of oral cancer which includes tongue, floor of mouth, gum or palate [27].

These days, two types of fillings, i.e. composite resin (CR) and glass ionomer cement (GIC), are gaining popularity due to aesthetic reason and biocompatibility. Compared to the conventional types of fillings, they have relatively lower densities between 1 g/cm^3^ to 2 g/cm^3^, approximately [28], [29]. This might be useful in reducing the undesirable impact of dental fillings on treatment planning.

A lot of work [26], [22], [23], [24], [30], [31], [32], [33] has been done to investigate the dosimetric impact of dental fillings, but they are mostly based on metal and amalgam fillings. On top of that, the majority of studies are centred on photon therapy. While Hu et al. [25] evaluated the SPR and spatial dose perturbation in the case of proton therapy, their samples consisted of commonly used materials, namely base metal, amalgam, zirconia and lithium disilicate. Thus far, none has looked into the effects caused by CR and GIC fillings on proton therapy treatment planning and delivery.

In view of this, the main goal of our work is to characterise the effect of CR and GIC fillings on proton therapy. Firstly, we will measure the SPR and compare it with the SPR estimated from the CT numbers in the CT image. The SPR from the CT scans will be estimated via two methods 1) SECT HLUT and 2) a commercial DECT-based SPR algorithm. This is to investigate if SECT or DECT is suitable for treatment planning directly without any material override. Secondly, we also aim to examine the dosimetric impact of dental fillings using proton beams with lower- and higher-energy spread out Bragg peaks (SOBPs).

## 2 Material and methods

### 2.1 Dental fillings

**Fig. 1:**
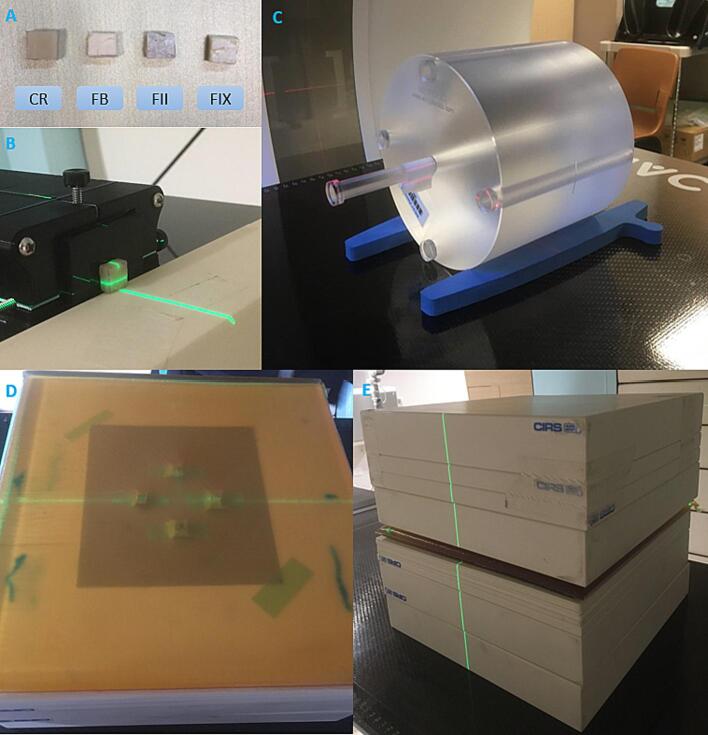
(A) Dental fillings made of composite resin (CR), Fuji Bulk (FB), Fuji II (FII) and Fuji IX (FIX). (B) R90 measurement with dental filling placed in the beam path. (C) CT scan setup of dental fillings along the central axis of PMMA CTDI phantom. (D) Setup of dental fillings below Superflab. (E) Measurement setup for dose perturbation of higher-energy proton beams, where plastic water phantoms were placed on top of the setup with dental fillings and Superflab shown in (D).

As shown in Fig. 1**A**, four types of dental fillings made of different materials - CR, Fuji Bulk (FB), Fuji II (FII) and Fuji IX (FIX) - were acquired from National Dental Centre Singapore (NDCS). The latter three belong to the class of GIC. The dental fillings were synthesized into a cuboid for this study. For each dental filling, the physical dimensions were measured at three different points using digital vernier callipers and averaged.

### 2.2 SPR measurements

The NCCS Hitachi Probeat proton therapy machine was used for the SPR measurement. This is a synchrotron accelerator with 98 discrete energy layers ranging from 70.2 to 228.7 MeV. Logos Ranger-300 (Logos Systems Int'l, USA) and BraggPeakView Software were used to measure the range at the 90 % distal fall-off of the integral depth dose profile (R90) for a single proton beam spot of 228.7 MeV and an in-air spot sigma of 2 mm at isocenter, with and without the dental fillings. The Ranger-300 device consists of a long cuboid of scintillation plastic. It was calibrated together with the vendor to ensure that the measured range in the device corresponded to the water equivalent range measured with a Bragg peak chamber in a water tank. After calibration, the device was used to measure the proton range in the dental fillings as shown in Fig. 1**B**. Three measurements with a re-setup were taken for each dental filling to account for positioning errors.

The average R90 values for each filling were then subtracted from that obtained without filling. This gave the water equivalent thickness (WET), which was then converted into water equivalent ratio (WER). **Equation** (1) shows the computation performed to determine WER, which will be referred to as the measured SPR hereafter.

(1) $$\mathrm{WER}=\frac{R90_{\mathrm{air}}-R90_{\mathrm{filling}}}{\mathrm{Physical}\;thickness\;}.$$

### 2.3 CT simulation

The dental fillings were placed along the central axis of a standard 16 cm diameter PMMA CTDI phantom (West Physics Consulting, GA, USA), as shown in Fig. 1**C**. CT scans were then taken using Siemens SOMATOM X.cite (Siemens Healthineers, Forchheim, Germany), with the scan and reconstruction parameters given in Table 1. For each filling, SECT and DECT scans were acquired, the latter comprising of TwinBeam (TB) DECT, and TwinSpiral (TS) DECT. 16-bit images were obtained for the SECT scans, with CT numbers up to the order of 60,000 HU. Each CT scan was reconstructed with and without applying iMAR algorithm. Even though CR and GIC fillings are not metallic, we included iMAR as an independent variable in our study to check if this algorithm, which is commonly applied for H&N cancer patients with metallic implants or crowns, would have any impact on the CT numbers of these fillings.

**Table 1:** CT scan and reconstruction parameters used. Abbreviations: SECT – single-energy computed tomography; TB DECT – TwinBeam dual-energy computed tomograpy; TS DECT – TwinSpiral dual-energy computed tomograpy; Au – gold; Sn – tin; iBHC – iterative beam-hardening correction; w/wo – with/without; iMAR – iterative metal artifact reduction.

CT Scanner	Siemens Healthineers SOMATOM X.cite		
	SECT	TB DECT	TS DECT
Tube Voltage	120 kVp	AuSn120	80/Sn150
Scan Mode	Single-energy CT	Dual-energy CT	
Field of View	500 mm		
Rotation Time	1 s	0.5 s	1 s
Pitch	0.8	0.45	0.55
Detector Collimation	128 x 0.6 mm	64 x 0.6 mm	
Slice Thickness	1 mm		
Slice Increment	1 mm		
Reconstruction Kernel	Qr40, Admire 3, iBHC Bone, w/wo iMAR		

For the pair of images (with and without iMAR) from the SECT scan, a rectangular region of interest (ROI) with an area of 6.29 mm^2^ was drawn on three consecutive slices, with the second slice corresponding to the middle plane of the filling. From these three ROIs, the mean and standard deviation of CT number for each filling were obtained. The SPR were then estimated applying our institutional HLUT (as shown in Figure S1 in the supplementary material) based on the mean CT number. Linear extrapolation was performed to obtain the corresponding SPR, since our institutional HLUT is clipped at 3000 HU, but the CT numbers of all four fillings were greater than 3000 HU.

For TB and TS DECT, the DirectSPR algorithm by Siemens was used to compute the SPR values. A research license was used for TB DECT as the clinical system does not support TB images at the moment. This algorithm leverages the varied attenuation properties of materials under different x-ray energies to extract more information regarding the elemental composition from a DECT scan, resulting in a more accurate SPR prediction [34]. These SPR images were then exported and evaluated using 3D Slicer [35]. Similar to SECT scans, the mean and standard deviation of SPR were calculated by drawing three rectangular ROIs of the same sizes inside the fillings in the SPR images. All six sets of SPR (from SECT, TB DECT and TS DECT, with and without iMAR) were then compared with the measured values for each filling.

### 2.4 Dose perturbation

Two SOBP plans were generated with RayStation v10A (RaySearch Laboratories AB, Stockholm, Sweden) using a CT-number-to-SPR HLUT, for lower-energy and higher-energy beams. The former used energies of 79.2 MeV to 133.7 MeV, with ranges of 4.90 g/cm^2^ to 12.70 g/cm^2^, forming a 7.8 cm SOBP, while the latter used energies of 130.2 MeV to 187.5 MeV, with ranges of 12.10 g/cm^2^ to 23.00 g/cm^2^, forming a 10.9 cm SOBP. This is to check if two different proton energy ranges typically used for H&N cancer treatment would result in different dose perturbation effects in a 4.5 mm thick dental filling (which is considered thick by clinical standard and serve as a worst-case scenario). The doses at the lower- and higher-energy SOBPs were 2 Gy and 3 Gy, respectively. Both used a field size of 10 x 10 cm.

Gafchromic EBT3 films were placed in between plastic water phantoms, at various depths from 0 cm to 3.0 cm. The depth of 0 cm corresponded to the position of the topmost film where the dental fillings were affixed, each placed 3.0 cm horizontally away from the crosshair along the two laser lines. Superflab (Radiation Products Design Inc., Albertville, United States) of 1.5 cm was then placed above the fillings (Fig. 1**D**). For lower-energy beams, a range shifter with a WET of 4.5 cm was used; for higher-energy beams, plastic water phantoms totalling 14 cm were placed on top of Superflab (Fig. 1**E**).

**Fig. 2:**
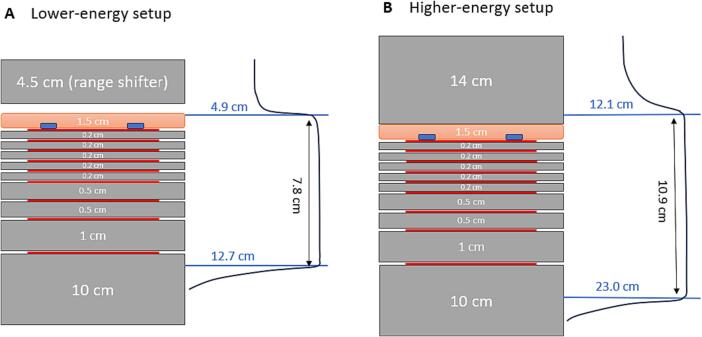
Schematics showing setups for dose perturbation measurement of (A) lower-energy and (B) higher-energy proton beams. The grey boxes represent plastic water phantoms of different thicknesses or range shifter, the orange layer represents Superflab, the blue rectangles represent dental fillings, and the red lines represent EBT3 films. The gap between the range shifter and Superflab in (A) depicts that the former is not in direct contact with the latter in the lower-energy setup. The corresponding SOBP positions are shown, with the beam penetration depths indicated in blue and SOBP widths indicated in black. NOTE: The schematics are not drawn to scale. (For interpretation of the references to colour in this figure legend, the reader is referred to the web version of this article.)

Fig. 2**A and 2B** depict the measurement setups for lower-energy and higher-energy beams, respectively. Taking the thickness of the dental fillings as 0.45 cm (average measured thickness of approximately 4.5 mm), the fillings were at 0.65 cm and 2.95 cm beyond the proximal edge of the lower-energy and higher-energy SOBP, respectively.

The exposed films were scanned using Epson Expression 12000XL (Epson America Inc., CA, USA) one day after exposure to ensure that the optical density growth had stabilized [36]. To reduce the systematic noise, which could arise due to variation in film response with distance from the scanner center [36] and presence of dust specks on the scanner, two scans were taken for each film, one being rotated 180° relative to the other. Although the longitudinal response was reported to be relatively stable [36], two extra scans were also taken for each film at a different position along the scan direction, similarly with one being rotated. This resulted in four scans in total for each film, where the average and sigma of dose perturbation ratio were obtained.

**Fig. 3:**
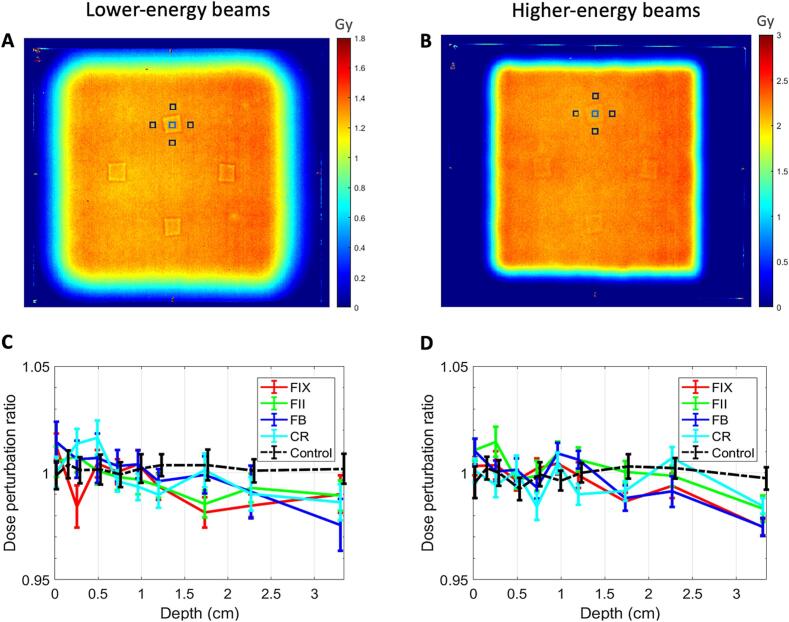
Examples of raw dose maps showing the dental fillings as four squares for (A) lower-energy and (B) higher-energy beams. The small blue square and the four small black squares around it denote the locations where the mean dose inside the filling and the mean reference dose, respectively, are obtained for one dental filling. Dose perturbation ratios of dental fillings and control at different depths for (C) lower-energy and (D) higher-energy beams. The error bars represent a 95% confidence interval around the mean values. FIX, FII, FB and CR denote Fuji IX, Fuji II, Fuji Bulk and composite resin, respectively. (For interpretation of the references to colour in this figure legend, the reader is referred to the web version of this article.)

Film calibration was done using doses from 0 Gy to 8 Gy. The red channel was selected to convert the scanned images into dose maps using MATLAB R2023a, due to its greater sensitivity to dose change in the lower-dose region (0 – 3 Gy) which corresponded to the dose range delivered from our plans. To reduce the effect of random noise, the dose maps were smoothened using a Gaussian-weighted moving average filter with a window length of 50. Dental filling locations were pinpointed, and dose perturbation ratios were calculated by dividing mean doses inside the filling by those in four similar regions outside the filling (reference) where no perturbation was expected, as illustrated in Fig. 3**A and 3B**.

This series of steps was repeated on films exposed without fillings in the setup. This constituted the control experiment to verify that the dose perturbation observed was indeed caused by the fillings instead of systematic dose profile variation.

Four Analysis of Variance (ANOVA) tests were performed, one for each filling, to check the null hypothesis that the means of dose perturbation ratios were equal at all depths. A two-tailed *p*-value of 0.05 was used to mark the significance of the test.

## 3 Results

### 3.1 Dental fillings

The physical dimensions of all four fillings are tabulated in Table 2. Each filling had an average lateral dimension and thickness of around 9.35±0.08 mm and 4.7±0.3 mm, respectively.

**Table 2:** Physical dimensions measured, water equivalent thickness (WET) and water equivalent ratio (WER) calculated (mean ± standard deviation) for each dental filling. The WER is the measured SPR to be compared with the SPR estimated from SECT and DECT scans.

Dental Filling	Length (mm)	Width (mm)	Thickness (mm)	WET (mm)	WER
Composite Resin	10.10 ± 0.05	9.58 ± 0.06	4.69 ± 0.10	7.88 ± 0.12	1.68 ± 0.06
Fuji Bulk	9.62 ± 0.09	8.57 ± 0.08	4.49 ± 0.08	7.94 ± 0.03	1.77 ± 0.04
Fuji II	9.54 ± 0.16	8.85 ± 0.07	4.79 ± 0.03	8.47 ± 0.08	1.77 ± 0.03
Fuji IX	9.44 ± 0.03	9.12 ± 0.13	4.66 ± 0.12	8.20 ± 0.11	1.76 ± 0.07

### 3.2 SPR analysis

**Fig. 4:**
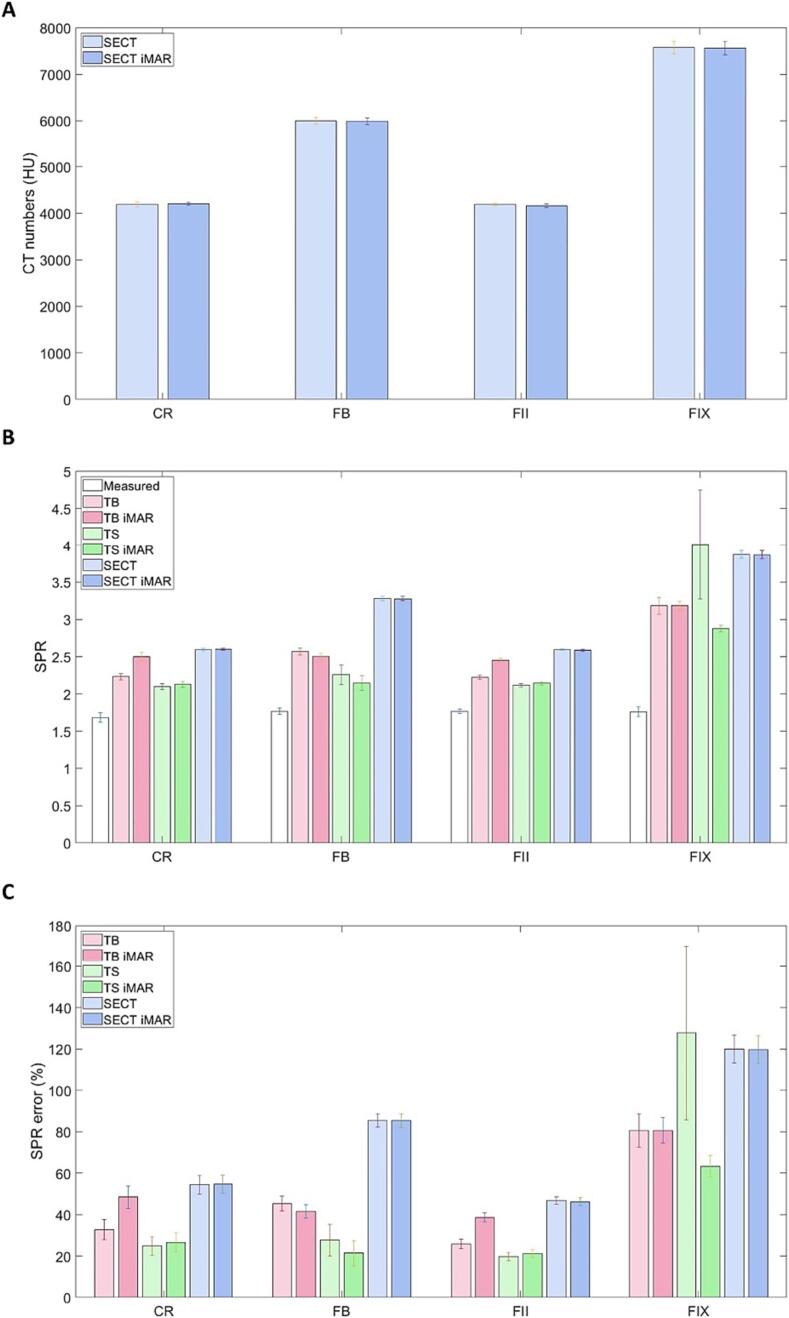
(A) CT numbers of dental fillings obtained from SECT and SECT iMAR. (B) Comparison of measured SPR with DECT-based DirectSPR (from TwinBeam (TB) and TwinSpiral (TS) scans) and SECT-based SPR applying the HLUT, for each dental filling. (C) Percentage error of DECT- and SECT-based SPR estimation. The error bars represent one standard deviation of uncertainties in (A) and (B), and the propagated error in (C). CR, FB, FII and FIX denote composite resin, Fuji Bulk, Fuji II and Fuji IX, respectively.

Table 2 shows the measured SPR for each dental filling, serving as the ground truth for comparison with SPR estimated from SECT and DECT scans. The measured SPR for all GIC fillings were close to 1.77, while CR had a slightly lower value at 1.68. Notably, all four fillings displayed similar measured SPR values despite their distinct CT number differences in SECT (Fig. 4**A**). CT numbers for all fillings exceeded 4000 HU, with FB and FIX exhibiting particularly high values of around 5000 HU and 7000 HU, respectively. Additionally, CT numbers measured with and without iMAR were similar, as indicated by the overlapping error bars.

Fig. 4**B** presents the SPR estimated from SECT, SECT iMAR, TB, TB iMAR, TS and TS iMAR as well as the measured values, while Fig. 4**C** illustrates the estimation errors**.** The conversion of CT number into SPR for SECT resulted in very dissimilar values from the measured SPR, with the percentage error ranging from 46.2 % (SECT iMAR – FII) to 119.9 % (SECT – FIX). This pointed to the unreliability of CT-number-to-SPR conversion with HLUT for an accurate SPR estimation. Overall, TS and TS iMAR yielded SPR closer to the measured values, with the lowest percentage error at 19.7 % (TS – FII). In contrast, their TB counterparts had a lower accuracy, where the lowest percentage error is 25.8 % (TB – FII). This might possibly be attributed to the better energy separation of TS compared to TB during CT simulation [34].

### 3.3 Dose perturbation

Fig. 3**A and 3B** are examples of dose maps at 2.0 cm depth before smoothening generated using MATLAB, for lower-energy setup and higher-energy setup, respectively. The squares on the dose maps represent the locations of the four dental fillings. The dose perturbation ratios calculated using these dose maps for each dental filling and the control at different depths (after accounting for the WET of EBT3 films (0.358 mm each [37])) were plotted in Fig. 3**C and 3D**. The values generally fluctuated around 1.0, but the deviations were seen to increase with depth. This was especially apparent at the depth of 3.0 cm for both setups. In comparison, the values for control remained relatively stable around 1 throughout all depths.

The subsequent ANOVA tests rejected the null hypothesis, with all the *F* values in the order of hundreds or thousands and *p*-values close to 0, hence attesting to the difference in all the dose perturbation ratios obtained. Nonetheless, the dose perturbation did not exceed 3 % for all the cases considered.

## 4 Discussion

This work marks the first attempt in characterising four types of dental fillings made of CR and GIC in terms of their impact on proton therapy. We quantified and compared the SPR obtained through measurement and estimations based on SECT HLUT and DECT DirectSPR algorithm, and evaluated the dose perturbation at different depths for lower-energy and higher-energy proton beams using GafChromic films.

As seen in Fig. 4**B**, the SPR obtained from both SECT and DECT were found to overestimate the true SPR for all fillings. The HLUT with SECT tends to give the worst result, as shown by the generally higher SECT and SECT iMAR bars in Fig. 4**B**. This is congruent with previous study by Longarino et al. [38], where the DECT-based SPR predictions of several dental fillings were found to be more accurate compared to SECT-based SPR predictions applying a HLUT. Nonetheless, despite improved SPR estimation using the DirectSPR algorithm, the difference still ranged from 19.7 % (TS – FII) to 127.7 % (TS – FIX). Thus, material override, guided by our SPR measurement results, becomes crucial for scenarios involving direct transversal of proton beam through substantial dental filling to reduce TPS dose calculation and range calculation error (which is supported by our TPS dose calculation results in Fig. S2-3), especially for higher-CT-number filling.

In our work, linear extrapolation was used for SPR estimation from the HLUT as the CT images are 16-bit [39]. In the case that CT number clipping was used instead, the SPR of all four fillings estimated from SECT and SECT iMAR would be approximately 2.15. This is much closer to the true values (1.68 to 1.77), but still deviating by around 21.5 % to 27.9 %. All these results underscored the insufficiency of current SPR estimation methods, hence warranting cautions when applying estimated SPR for proton treatment planning.

Regardless of the proton beam energies used, the dose perturbation was observed to be 3 % maximum. The dose perturbation was most pronounced at the largest depth along the beam path. Compared to the reported values of around 20 % by Hu et al. [26], the perturbation observed with CR and GIC fillings was much less. This suggested that the use of CR and GIC fillings are much more proton compatible compared to the commonly used metal or amalgam fillings; any mis-contouring, mis-assignment of materials are less detrimental to the dose calculation accuracy compared to metal or amalgam fillings.

In this study, a small dose perturbation was observed, contrasting with a rather large SPR deviation, which may seem paradoxical. In fact, these two are not directly correlated; dose perturbation relies on material thickness, whereas SPR deviation reflects the accuracy of SPR prediction using SECT and DECT scans, independent of thickness. SPR estimated from the SECT-based HLUT or the DECT algorithm are only accurate for tissue-equivalent material and the accuracy is usually not guaranteed for non-biological material.

In a clinical situation where H&N patients with dental fillings are encountered, the impact of filling on the dose calculation accuracy could be confounded by the complex and heterogeneous geometry in the region, including the teeth, bone and nasal cavities. Additionally, the filling thickness of a real patient is expected to be smaller. Therefore, the results reported here could have been an exaggerated version of a realistic scenario. The use of an anthropomorphic phantom might be able to provide better clinical practicality, and will be an interesting aspect to explore as a future work.

## Author contribution statement:

Study conception and design: Hong Qi Tan.

Data acquisition and analysis: Yun Ming Wong, Calvin Wei Yang Koh, Clifford Ghee Ann Chua, Kah Seng Lew, Ping Lin Yeap, Hong Qi Tan.

Data interpretation: All authors.

Statistical analyses: Yun Ming Wong, Hong Qi Tan.

Obtained funding: Hong Qi Tan.

Administrative, technical, or material support: Calvin Wei Yang Koh, Kah Seng Lew, Hong Qi Tan.

Study supervision: Wen Siang Lew, James Cheow Lei Lee, Sung Yong Park.

Drafting of manuscript: Yun Ming Wong.

Approval of final manuscript: All authors.

## Funding support

Hong Qi Tan is supported by the Duke-NUS Oncology Academic Program Goh Foundation Proton Research Programme (08/FY2021/EX(SL)/92-A146), Clinical & Systems Innovation Support – Innovation Seed Grant (08/FY2022/P2/02-A68).

## CRediT authorship contribution statement

**Yun Ming Wong:** Software, Formal analysis, Investigation, Data curation, Writing – original draft, Visualization. **Calvin Wei Yang Koh:** Formal analysis, Investigation, Methodology. **Kah Seng Lew:** Investigation, Methodology. **Clifford Ghee Ann Chua:** Investigation. **Ping Lin Yeap:** Investigation. **Wibawa Andrew:** Investigation. **Master Zubin:** Investigation. **Sharon Shuxian Poh:** Resources. **Wen Siang Lew:** Supervision. **James Cheow Lei Lee:** Supervision. **Sung Yong Park:** Supervision. **Hong Qi Tan:** Conceptualization, Methodology, Software, Investigation, Writing – review & editing, Project administration, Funding acquisition.

## Declaration of competing interest

The authors declare that they have no known competing financial interests or personal relationships that could have appeared to influence the work reported in this paper.
