# Supplementary material for: Effects of modern aesthetic dental fillings on proton therapy
Source: Phys Imaging Radiat Oncol. 2024 Feb 15;29:100552. doi: 10.1016/j.phro.2024.100552 (PMC10891317; doi:10.1016/j.phro.2024.100552)
Supplement: Supplementary data 1 [file mmc1.docx]

Supplementary materials

**Glossary of Abbreviations**

CT: computed tomography

GIC: glass ionomer cement

HLUT: Housfield look-up table

iMAR: iterative metal artifact reduction

SECT: single-energy computed tomography

SPR: stopping power ratio

**Figure S1.** Our institutional HLUT for SECT-based CT-number-to-SPR conversion.


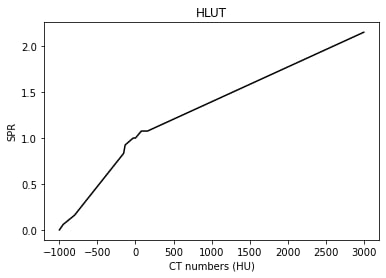


**Figure S2.** Dose profile calculated using treatment planning system for lower-energy proton beam passing through (A) composite resin, (B) Fuji Bulk, (C) Fuji II, and (D) Fuji IX. Reference denotes the dose calculated without dental fillings in path. iMAR (Measured) and iMAR (SECT) denote the dose calculated on images reconstructed using iMAR, with dental fillings’ SPR overridden with measured values and not overridden, respectively. Non-iMAR (Measured/SECT) denote the corresponding dose calculated on images reconstructed without iMAR.


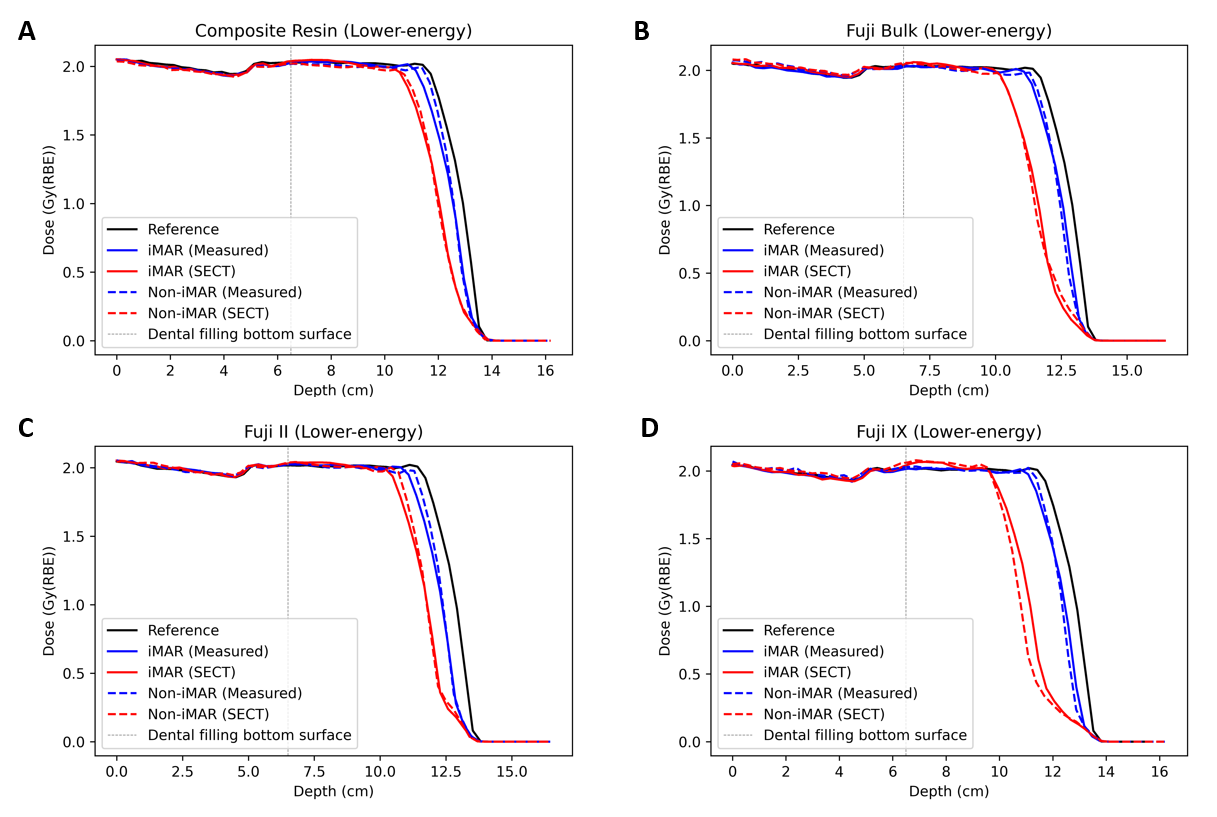


**Figure S3.** Dose profile calculated using treatment planning system for higher-energy proton beam passing through (A) composite resin, (B) Fuji Bulk, (C) Fuji II, and (D) Fuji IX. Reference denotes the dose calculated without dental fillings in path. iMAR (Measured) and iMAR (SECT) denote the dose calculated on images reconstructed using iMAR, with dental fillings’ SPR overridden with measured values and not overridden, respectively. Non-iMAR (Measured/SECT) denote the corresponding dose calculated on images reconstructed without iMAR.


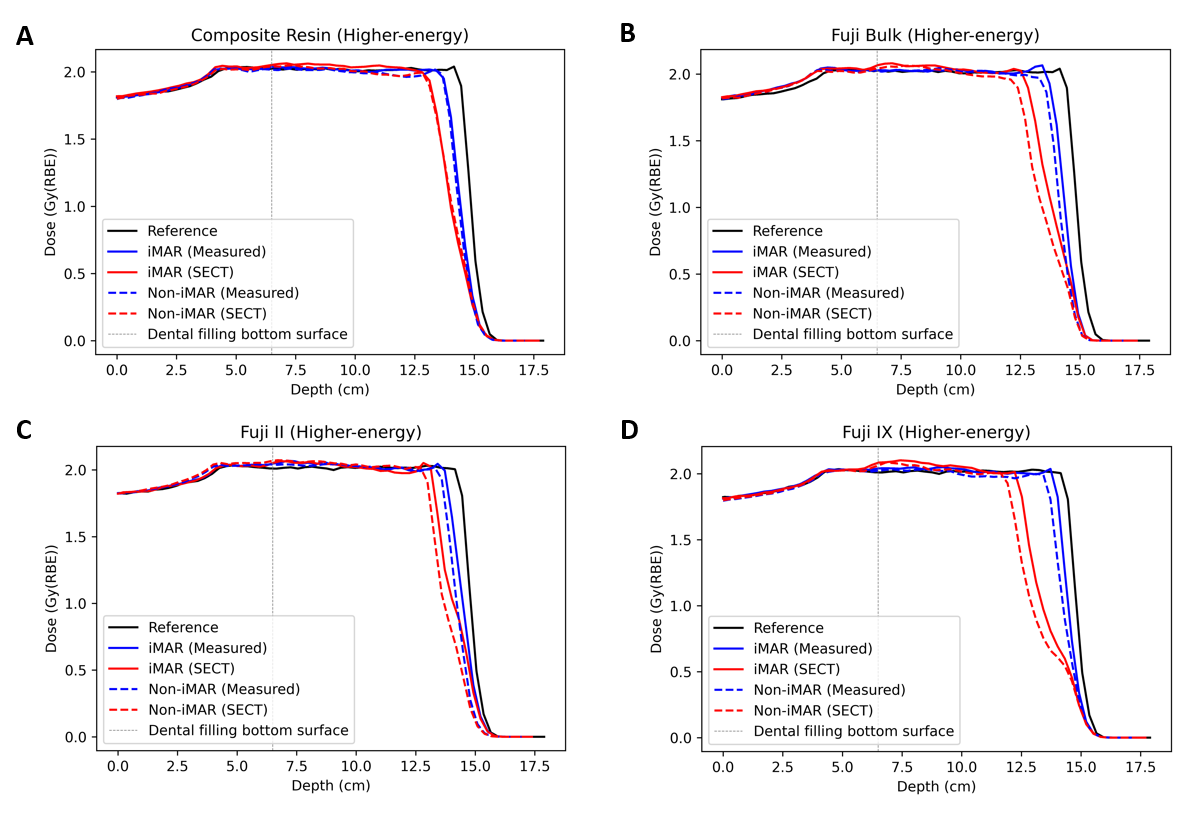


**Additional comments:**

As reported by Khiri et al., the density of GIC sample increases with ageing time [29]. A relevant topic for future study is to investigate how the ageing time of GIC fillings during the manufacturing process would affect their SPR and dose perturbation effect. Certainly, there is an interplay between the density and the desirable mechanical properties for its durability, hence the ageing time should ideally be tuned such that the filling poses the least impact on proton therapy while remaining wear-resistant. Nevertheless, the composition of GIC fillings investigated in this study was similar to those being used clinically, hence they should serve as a practical prototype to illustrate the effect on proton treatment planning and delivery at present.
